# Tissue engineering of mouse uterus using menstrual blood stem cells (MenSCs) and decellularized uterine scaffold

**DOI:** 10.1186/s13287-021-02543-y

**Published:** 2021-08-23

**Authors:** Nouri Arezoo, Hajian Mohammad, Monsefi Malihezaman

**Affiliations:** Cellular-Developmental Biology Lab, Biology Department, College of Sciences, Shiraz University, Adabiate St., 71456-85464 Shiraz, Fars Iran

**Keywords:** Decellularization, Menstrual blood stem cells, Recellularization, Uterine scaffold

## Abstract

**Background:**

Uterine tissue engineering can provide the opportunity for curing female infertility. Natural scaffold is a good choice to recapitulate the architecture and functionality of the native tissue. In this study, we purposed the potential of uterine decellularized scaffolds as an adequate natural niche for MenSCs differentiation toward uterus-specific cell lineages.

**Methods:**

Mouse’s uterus was decellularized by immersion of hypo and hypertonic salts or freeze–thaw cycle followed by immersion in Triton X-100 and SDS solutions. MenSCs were isolated from the menstrual blood of 6 healthy women. The decellularized and recellularized samples were prepared for further in vitro and in vivo analyses.

**Results:**

Histochemical studies and Raman spectroscopy revealed uterine ECM was preserved well, and the cells were completely removed after decellularization. Scanning electron microscopy (SEM) showed that the 3D ultrastructure of the uterus remained intact. Flowcytometric examination with CD34, CD44, CD105, CD106, CD144 markers revealed stem cell characters of cells that isolated from menstrual blood. MTT assay confirmed the bioavailability of MenSCs cultured scaffolds after 7 and 10 days.

**Conclusion:**

Histochemical studies, SEM images, and Raman spectra showed MenSCs seeded and growth in uterine scaffolds. Immunostaining using anti-cytokeratin (CK), anti-desmin (Des), anti-vimentin (Vim), and anti-smooth muscle actin (SMA) antibodies showed MenSCs differentiation to epithelial and smooth muscle tissues. The Raman spectroscopy revealed the extracellular matrix (ECM) of decellularized uterine scaffolds was well preserved. The decellularized uterus can be considered a promising vehicle to support cell transplantation and differentiation. MenSCs are a good choice for uterine tissue engineering. The complete decellularization from mice uterine tissue was done by combining chemical agents

## Background

Many diseases cause damage to tissues or organs and destroyed their functions. Also, a total of 122,682 fatal traffic injury cases were registered during 2011–2017. The annual mortality rates for road traffic fatalities were 21.9 and 5 per 100,000 people [1]. Transplantation is the only mode of therapy to replace organ failure affecting such as kidneys, liver, heart, lungs, and pancreas. High cost, low number of qualified donors, the risk of rejection, and side effects of immunosuppressive drugs are organ transplantation problems. Approximately 50% of all transplanted organs are rejected within 10–12 years. This rate is varied for different organs and the old of recipients. So, there is a great need for better ways to reduce or eliminate organ rejection.

Tissue engineering is an important field of regenerative medicine for tissue repair. In this method the use of stem cells such as mesenchymal stem cells (MSCs) deriving from a different source of body and scaffold improve or replace damaged tissues. Scaffolds used in tissue engineering are divided into two groups of natural and synthetic scaffolds. Natural scaffolds can provide an adequate environment similar to that of the extracellular subunit material for the cells, and due to the proper immunological response, angiogenicity, the ability to improve cell adhesion and homeostasis are very important [2].

A rich source of stem cells will be obtained from menstrual blood (Menstrual Blood Stem Cells: MenSCs). During the past decade the abundance, easy access, periodically and non-invasively collection, the ability to regularly donated, and autologous transplantation are the reasons that these cells are the ideal source for tissue engineering. So far, many cases of extraction and differentiation of MenSCs have been reported in various cell types. MenSCs have shown positive therapeutic potential for the treatment of various diseases. The ability to differentiate cells extracted from menstrual blood into skeletal muscle cells for the treatment of Duchenne muscular dystrophy has been reported in a mouse model [3]. MenSCs promote both regenerations of myocardial damage and the improvement of cardiac function [4]. Treatment for injuries caused by stroke was shown by the effective differentiation of MenSCs to functional neural cells [5]. MenSCs were differentiated into liver cells [6]. Menstrual-derived stem cells are differentiated into cartilage and replaced with nano-fibrous scaffolds [7]. To differentiate stem cells from menstrual blood to epidermal cells, a culture method of keratinocytes isolated from the foreskin of neonates of 2–10 months old boys was used. These cells were introduced as a usable and non-invasive source for differentiation into epidermal cells in the treatment of skin lesions [8].

The uterus is responsible for the main task of pregnancy and fetal development. Hysterectomy is caused by some diseases or injuries. In the present study, we attempted to construct a uterus using a natural remodeled scaffold of the mouse decellularized uterus and MenSCs.

## Materials and methods

### Animals

A total of eight mature female Balb/c mice weighing between 32 and 38 g were obtained from the Animal House of Shiraz University of Medical Sciences, Shiraz, Iran. The mice were adapted to the laboratory conditions for two weeks before the experiments. Animals were kept at a controlled temperature (22–25 °C) and under a 12 h light–dark cycle (lights on from 6:00 until 18:00) with free access to food and tap water. The animal experiments were approved by the Institutional Animal Ethics and Health Committee of the Biology Department of Shiraz University (IR.SUSC.1397.S9431201) and were performed according to the principles of the care and use of laboratory animals established by the National Institute of Health.

### Preparation of decellularized scaffolds

Uterine tubes were removed and divided into 1 cm pieces. Decellularization was performed using three methods: (1) The samples were immersed into (SDS) Sodium Dodecyl Sulfate (Merck, Germany) and triton X-100 solutions (Merck, Germany) at concentrations of 0.1, 0.3, 0.6%, each for 24 h. (2) the samples were stored at 0 °C (freezing) for one week and then melted by transferring to room temperature (37 °C). The specimens were subsequently immersed into the 0.5% SDS and Triton X-100 0.1% each for 48 h. (3) The samples were immersed in NaCl solutions at concentrations of 1.5, 3 and 5 mol each for 24 h, and then immersed into SDS and triton-X100 similar to the second method.

### Collection of menstrual blood

The MenSCs were isolated from 6 healthy volunteers of fertile women aged between 20 and 40 years. The blood donation was approved by Ethics and Health Committee of the Biology Department of Shiraz University (IR.SUSC.1398.S943120-CONSENT FORM). The experimental procedures for menstrual blood samples and MenSC were carried out in accordance with the approved guidelines. We received informed consents from all of the donors, and all the donors provided consent for the use of their menstrual blood samples in scientific research. About 4 ml of menstrual blood (MB) was collected using two methods: (1) using Diva Cups (European Lady Cup) that were inserted into the vagina for a mean of 3 h (2–4 h) on the second day of the menstrual cycle (2) using sterile collection containers. MB was transferred into falcon tube containing 4 ml PBS/EDTA 0.5 mM, 40 µl Penicillin–Streptomycin (Shell max, Iran), and 8 µl Amphotericin B (Gibco, USA) 250 µg/ml.

### Isolation and culture of MenSCs

Mononuclear cells were separated using ficoll (Inno-train, Germany) density gradient centrifugation and washed out in PBS. Then, the cell pellet in the tube was suspended in DMEM-F12 (Bio-IDEA, Iran) medium supplemented with 10% FBS and cultured in tissue culture plates. The cells were kept in 37 °C incubator with 5% CO_2_. After removal of non-adherent cells on the second day of incubation, the culture of adherent cells continued until 70% confluency.

In direct culture, 5 ml MB were cultured in 25 ml flask with 10 ml DMEM-F12 containing 10% FBS, 1% Pen/Strep, and 0.1% Amphotericin B in 37 °C incubator with 5% CO_2_ for 5 days. The red blood cells were washed and the culture of adherent cells was continued until up to 70% confluency. The stem cells were passaged 4–6 times.

### Flowcytometry

The harvested cells were fixed with paraformaldehyde. The cells were washed and the non-specific binding sites were blocked by PBS containing gout serum. The monoclonal FITC conjugated anti CD144, CD34, CD44, CD 106, and CD 105 antibodies (Sigma, USA) were added and incubated at 4 °C for an hour. The cells were centrifuged in cold PBS at 2100 rpm for 5 min. The supernatant was removed and the cells were measured in the FL1 channel of flowcytometer (BD Company, USA). The percentages of the cells that reacted to CD markers were analyzed by histogram using the WINmdi 2.9 software.

### Recellularized scaffolds

The decellularized scaffolds were divided into 5 mm pieces. They were cut longitudinally by insulin needles and sterile pins. Samples were sterilized with 0.1% peracetic acid (Merck, Germany) for 10 min then washed with sterile PBS (Shell max, Iran) and exposed to UV radiation for 20 min under the laminar hood. The total number of 2 × 10^4^ MenSCs were added to each scaffold and incubated in a 96-well culture plate at 37 °C and 5% CO_2_. After 30–40 min, 200 µl DMEM/F12 containing FBS 10% and pen/strep 0.1% was added and cultured for 2 weeks. The media were changed every 24 h. The co-culture of scaffolds and MenSCs were checked daily. The photographs were taken by a digital camera (Nikon, Japan).

### Histological studies

Decellularized scaffolds were fixed in a 10% buffered formalin solution for 1 week then paraffin blocks were prepared [9] and 5 µm thick sections stained with hematoxylin–eosin (H&E) and Masson’s trichrome staining. The histological changes of scaffolds such as cell removal, normal arrangement and structure of collagen fibers were evaluated microscopically.

Recellularized scaffolds were fixed in 10% buffered formalin in a 96-well culture plate for 2 h. Scaffolds were prepared histologically and 4 µm thick sections stained with hematoxylin–eosin and hoechst (American Sigma) staining. Photomicrographs were prepared by a digital camera (Nikon, Japan).

### MTT assay

To evaluate the MenSCs bio-viability and growth in the scaffolds after 7 and 10 days MTT colorimetric assay was applied. Briefly, the MTT solution was prepared at 1 mg/ml in PBS and was filtered through a 0.2 µm filter. Then scaffolds were washed with PBS and MTT solution at 1:10 of total volume was added to each scaffold in 96-well culture plate. Tissues were incubated for 4 h at 37 °C with 5% CO_2_ and complete humidity. After 4 h, the MTT solution was removed and replaced with 200 µl of DMSO. The plate was further incubated for 2 h at room temperature, and the optical density (OD) of the wells was determined using an Eliza reader (Bmg Labtech, Germany) at a test wavelength of 570 and 630 nm. Percent of the cell viability was calculated using the equation: (mean OD of treated cells/mean OD of control cells) × 100.

### Scanning electron microscopy

To evaluate the microarchitecture of the decellularized scaffolds and nesting cells after recellularization, scanning electron microscopy (SEM) was performed. The supernatant of each recellularized scaffold was removed and washed with PBS 2–3 times. Samples were fixed with glutaraldehyde 4% (Merck, Germany) for 1.5 h and washed 2–3 times with PBS. The specimens were dried with a freeze dryer (LD Plus Alpha 2–4, Christ, Germany) and were covered with gold using Sputter Coater (Q150R-ES: Quorum Technologies, England). The photomicrographs were imaged by TESCAN-Vega 3 microscope (TESCAN, Czech Republic). To analyze the structural or chemical properties of the sample, an Energy Dispersive X-Ray Spectrometer (EDS) spectrometer was used.

#### Immunohistochemical (IHC) studies

To evaluate the differentiation of MenSCs, the recellularized scaffolds were fixed in 4% paraformaldehyde for 24 h followed by immersing in 30% sucrose as a cryoprotectant for 72 h; at the end, they were transported to the liquid nitrogen. The tissues were then embedded in paraffin and sectioned at a thickness of 5 µm. Endogenous peroxidase was neutralized by 0.3% H_2_O_2_ in methanol, and non-specific binding sites were blocked by 4% goat serum in PBS. The samples were incubated with anti-cytokeratin (AE1/AE3), anti-desmin (D33), anti-vimentin (SP20), and anti-smooth muscle actin (1A4) antibodies all from Biocare Medical Company and using Detection kit (Master Diagnostic) overnight at 4 °C. Then, they were washed with PBS three times for 15 min. Finally, the samples were treated with streptavidin–horseradish peroxidase complex followed by incubation with diaminobenzidine (DAB, Dako) and counterstained with hematoxylin.

#### Confocal Raman spectroscopy

To detect the extracellular matrix preservation of decellularized scaffolds, samples were placed on a 1 mm thick quartz window. Raman spectral mapping was recorded for tissue areas of 4 × 4 mm. Raman spectra were collected using an inverted optical microscope (Lab Ram HR, Horiba, Japan) equipped with a 50×/0.50 NA objective. A 785 nm excitation laser, with the power of 100 mW at the sample, was used for excitation. Raman maps were measured by raster-scanning the movable stage (Duo Scan) is equipped to allow spectral imaging. The integration time at each pixel was 1 s. Auto-fluorescence images of the tissue samples were recorded for the same areas using SWIFT Fast confocal imaging integrated into the Raman microscope. The excitation was based on a laser at 532 nm (10 mW), and detection in the range of 532–600 nm.

Spectra were recorded in the range of 400–1800 cm^−1^.

#### Statistical analysis

Statistical analysis of the data was confirmed with SPSS software and normal data with Kolmogorov–Smirnov test confirmed with high confidence. Then, One-way ANOVA was performed at *p* < 0.05. The Tukey post hoc test was used to obtain the mean and standard deviation for each parameter. The graphs were plotted using EXCEL software.

## Results

**Fig. 1:**
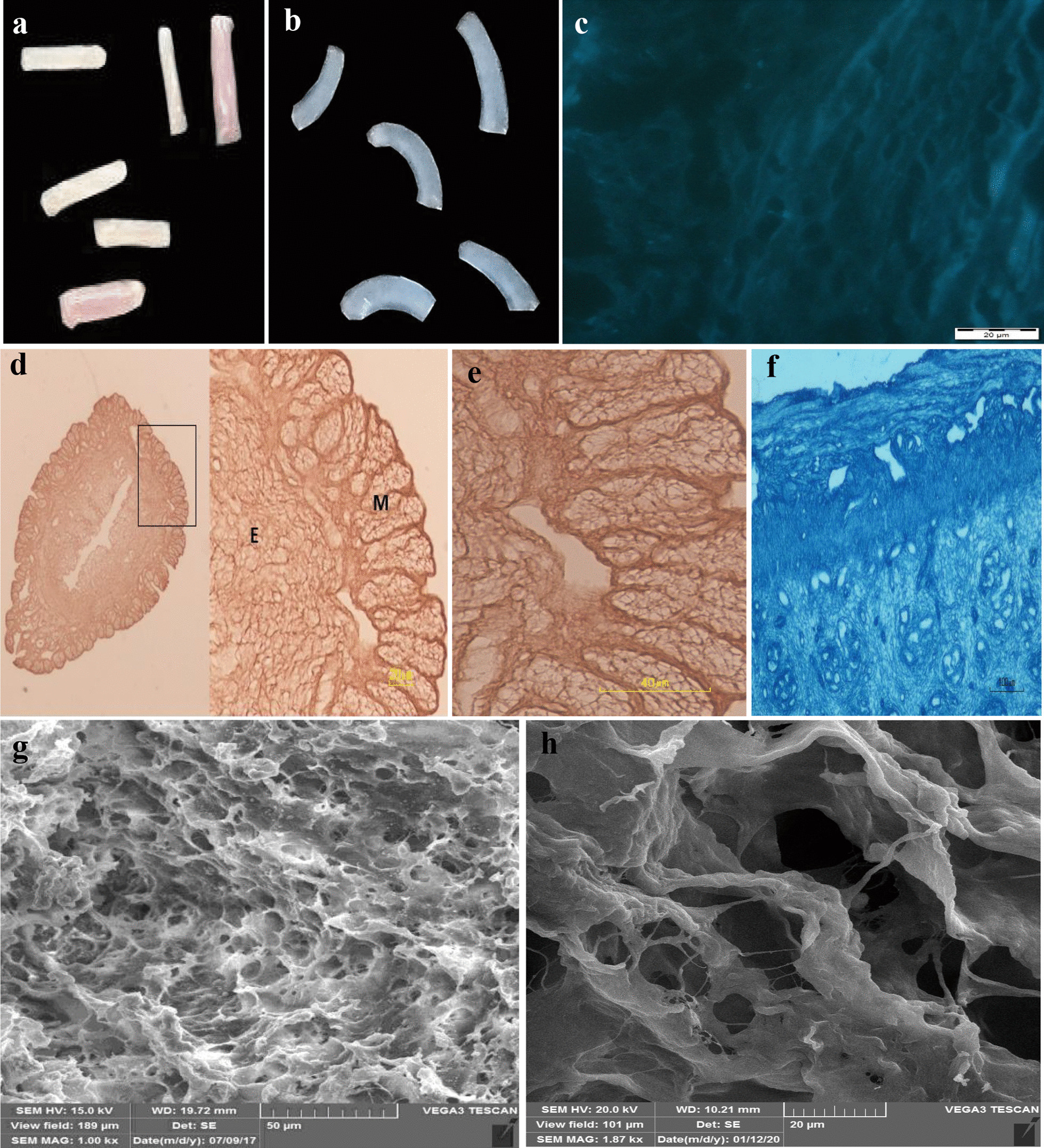
Tissue sections of the decellularized uterus using SDS and Triton-X100. Uterine segments before (**a**) and after decellularization (**b**). Tissue section of the decellularized uterus; hoechst staining (**c**), hematoxylin and eosin staining (**d**) higher magnification of myometrium (**e**), and its Masson’s trichrome staining (**f**). Micrograph of decellularized scaffolds (**g** and **h**) with different magnifications. The intact arrangement of collagen fibers was showed in **h**. E: endometrium, M: myometrium

The gross examination of the decellularized uterus with all methods revealed a whitish translucent appearance and confirmed decellularization efficacy (Fig. 1a, b). The usage of different concentrations of SDS and Triton X-100 solutions revealed cell removal along with the intact ECM architecture after H&E (Fig. 1d, e) and Masson’s trichrome staining (Fig. 1f). The vasculature framework without endothelial cells and ECM around the endometrial gland were preserved in the decellularized samples. There were some remnant nuclei in the endometrium of the decellularized uterus after two other methods of hypertonic and hypotonic solutions and freeze and thaw. Therefore, natural scaffold preparation using SDS and Triton X-100 was selected for MenSCs recellularization.

Hoechst staining confirmed the uterine decellularization except for the presence of a very few nuclei residues in some areas of the microscopic field (Fig. 1c). The SEM photomicrograph confirmed the intact uterine three-dimensional ECM architecture without any cell or nucleus component (Fig. 1g, h).

**Fig. 2:**
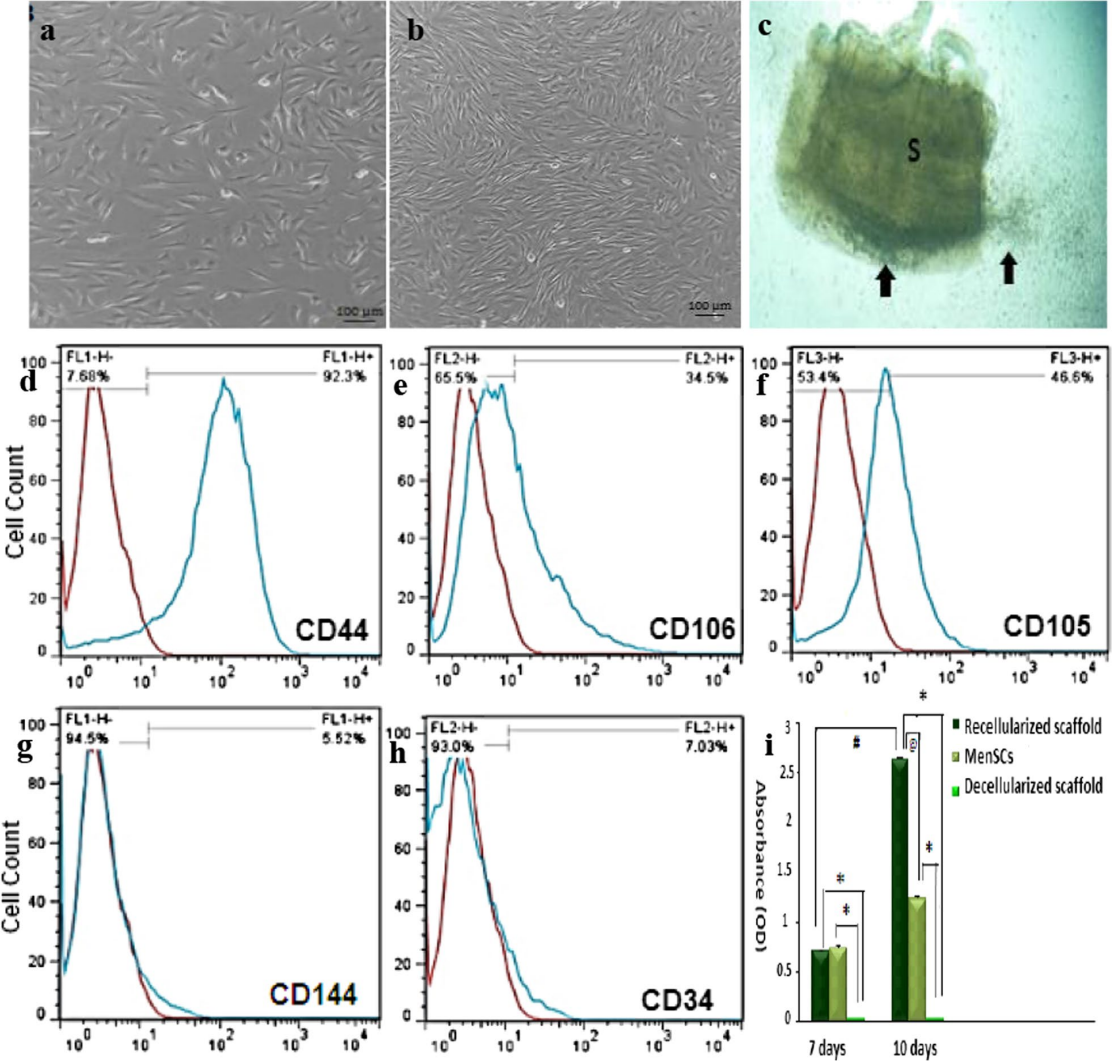
The second passage of Menstrual blood stem cells (MenSCs) culture extracted by ficoll method (**a**) and by direct culture method (**b**). MenSCs migration to the decellularized scaffold after 7 days of culture (**c**). Flowcytometry of MenSCs for CD44 (**d**), CD 106 (**e**) and CD 105 (**f**) markers showed 92.3%, 34.5% and 46.6% overlapping respectively. MenSCs did not detect CD144 as an endothelial cell marker (**g**) and CD34 as a hematopoietic stem cell marker (**h**), 5.52% and 7.03% respectively. The MTT assay revealed higher viable cells of MenSCs culture after 10 days when compared to 7 days culture (**i**)

The isolated MenSCs were adherent to the culture plates after 24 h. The proliferation rate of MenSCs in the direct culture method was faster than the ficoll usage method. By reaching 80% confluency after 3 days, the cells exhibited spindle-shaped morphology like fibroblasts (Fig. 2a, b). Flowcytometric analysis revealed the expression of mesenchymal markers such as CD44, CD 106, and CD 105 (Fig. 2d–f). Further analysis showed the lack of hematopoietic stem cells (CD34) and endothelial cells (CD144) markers (Fig. 2g, h). The immunophenotypic analysis showed that MenSCs were positive for CD44 (92.3%), CD 106 (34.5%) and CD 105 (46.6%) and negative for CD144 (5.52%) and CD34 (7.03%).

In vitro study showed MenSCs migrated and entered the decellularized scaffold. The highest MenSCs migration into the decellularized scaffold was after 72 h. After one week, MenSCs penetrated the scaffold completely and there were a few numbers of adherent cells in the monolayer culture plate (Fig. 2c).

MTT assay revealed that the decellularized scaffold was non-toxic and MenSCs were seeded into the decellularized framework of each three layers of the uterus. The cell viability was compared with that in monolayer culture after 7 and 10 days. As time passed, proliferation and viability significantly increased. The MenSCs viability was significantly lower in the cells seeded on the scaffold after 7 days compared to those seeded in 10 days (Fig. 2i).

**Fig. 3:**
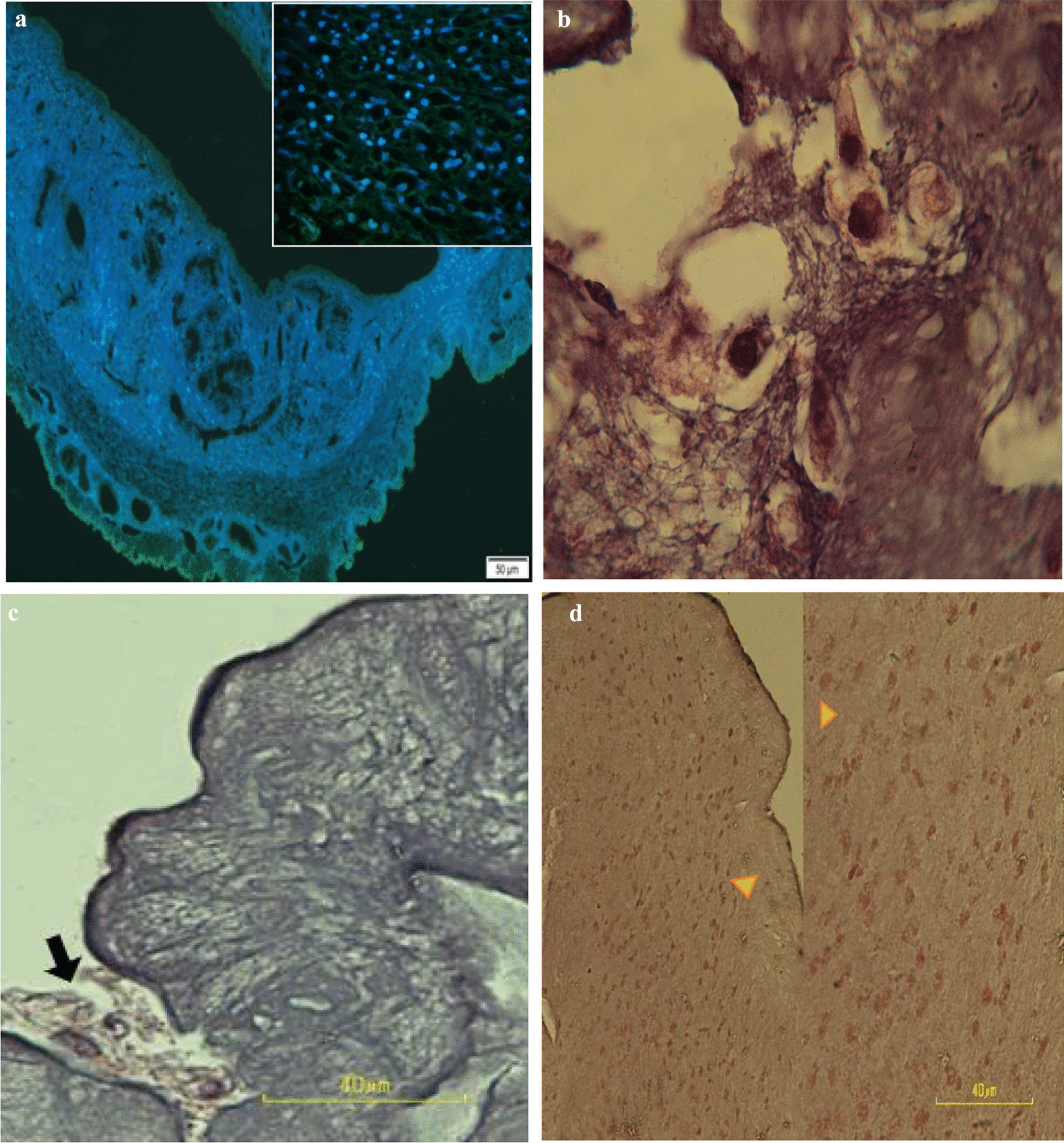
Tissue sections of recellularized scaffolds (**a**) after Hoechst staining. The light blue spots showed the live cell nuclei. Tissue sections of the recellularized scaffold after 7 days (**b**, **c**) and 10 days (**d**) of MenSCs culture. Please note the cell migration between collagen fibers of the uterine scaffold in higher magnification (arrowheads). Hematoxylin and eosin staining

**Fig. 4:**
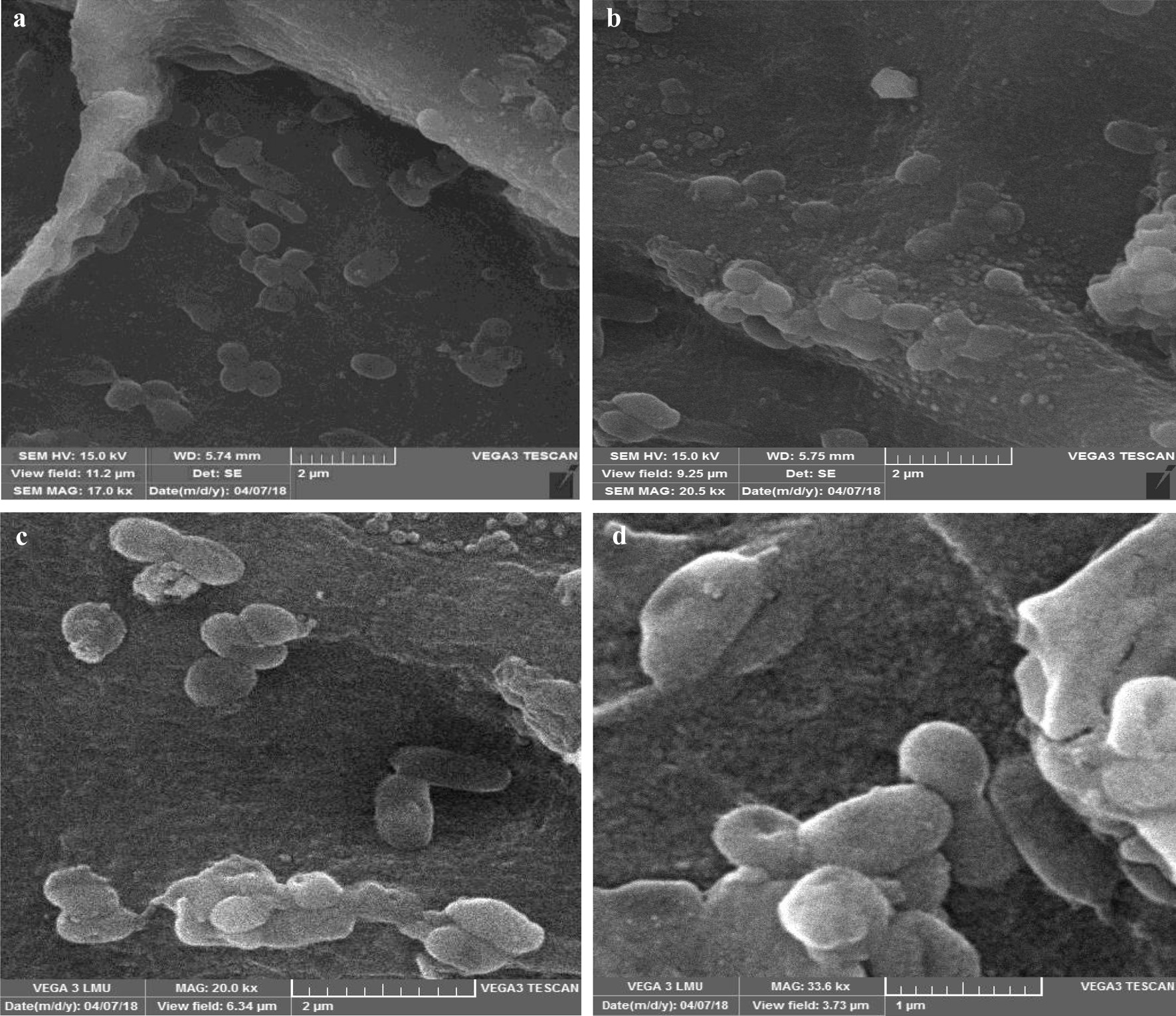
Micrograph of recellularized scaffolds with different magnifications. Please note some spherical or oval cells and short processes of differentiated cells

A microscopic study revealed the MenSCs in the recellularized scaffolds were distributed with different phenotypes in all layers of the uterus. The cells located in the myometrium had spherical with round or oval nuclei (Fig. 3a). The cells scattered within the glandular framework of the endometrium were fibroblast-like structures with oval pale nuclei. Therefore, it seems that the ECM architecture and content regulated the cell morphology. The attached cells to the surface of the perimetrium were squamous in shape (Fig. 3b–d). Hoechst staining also showed the presence of MenSCs nuclei as bright blue nuclei in the recellularized scaffold is due to specific binding of the dye in a small groove between the two strands of DNA (Fig. 3a). SEM photomicrographs confirmed the migration of different cell phenotypes according to the location within the scaffolds (Fig. 4a–d).

**Fig. 5:**
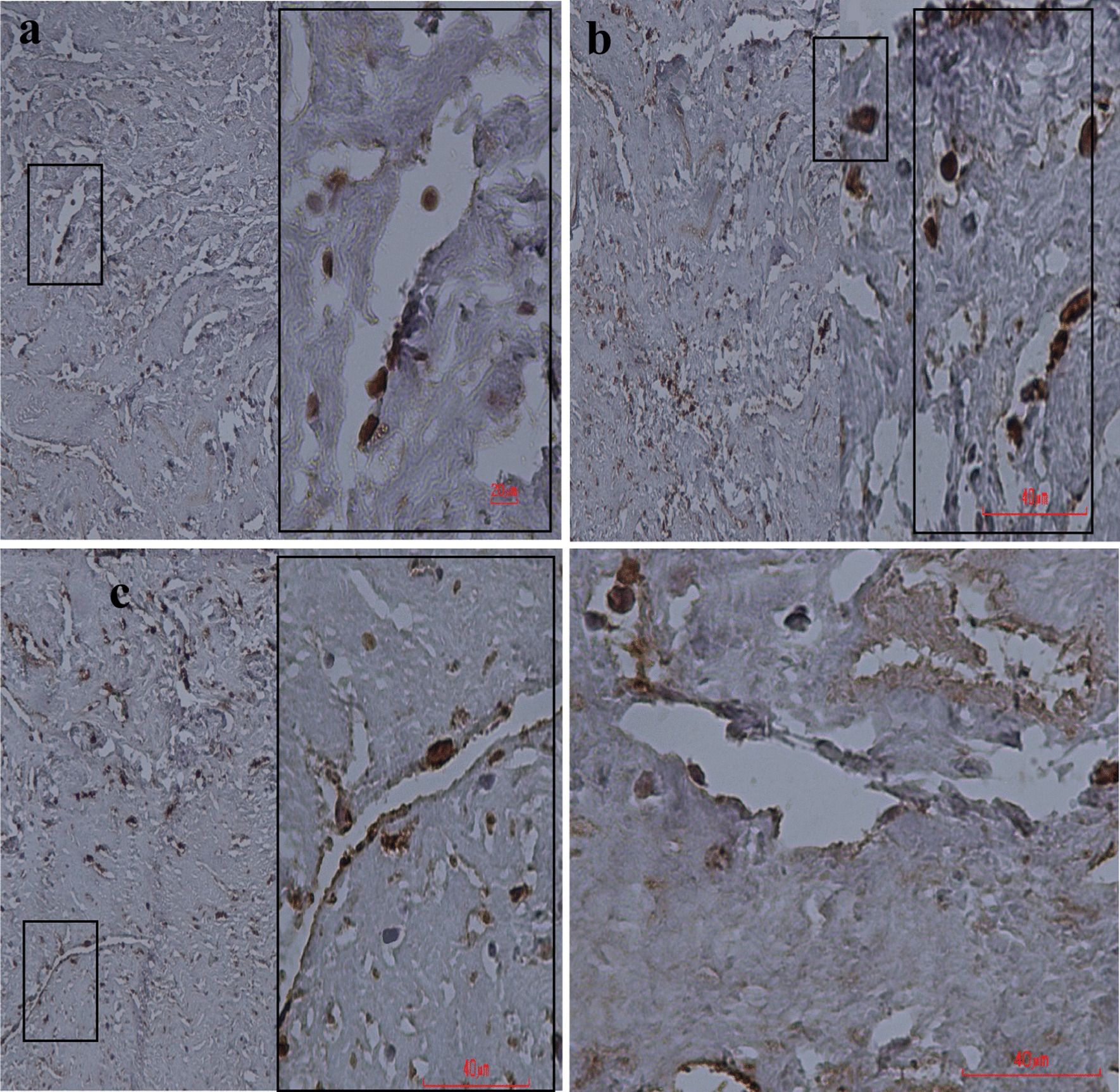
The immunohistochemical staining of the recellularized uterine scaffold. Please note the positive reaction of migrant cells as brown dark nuclei stained (**a**) by anti-cytokeratin (CK), **b** anti-smooth muscle activin (SMA), **c** anti-vimentin (Vim), and **d** anti-desmin (Des) antibodies against violet nuclei (arrowhead). Scale bar = 40 µm

Immunostaining showed that seeded cells within the endometrium, myometrium, and perimetrium of the decellularized uterine scaffolds have the potential for differentiation to different cell types. Anti-cytokeratin (CK) antibody stain the acidic and basic (Type I and II) subfamilies of cytokeratins in human epithelia, anti-smooth muscle actin (SMA) show the presence of smooth muscle cells in vessel walls and myometrium and anti-desmin and anti-vimentin antibodies detect intermediate filament of smooth muscle. Therefore, the positive reactions with these antibodies revealed the differentiation of MenSCs to the epithelium and smooth muscle (Fig. 5a–d).

**Fig. 6:**
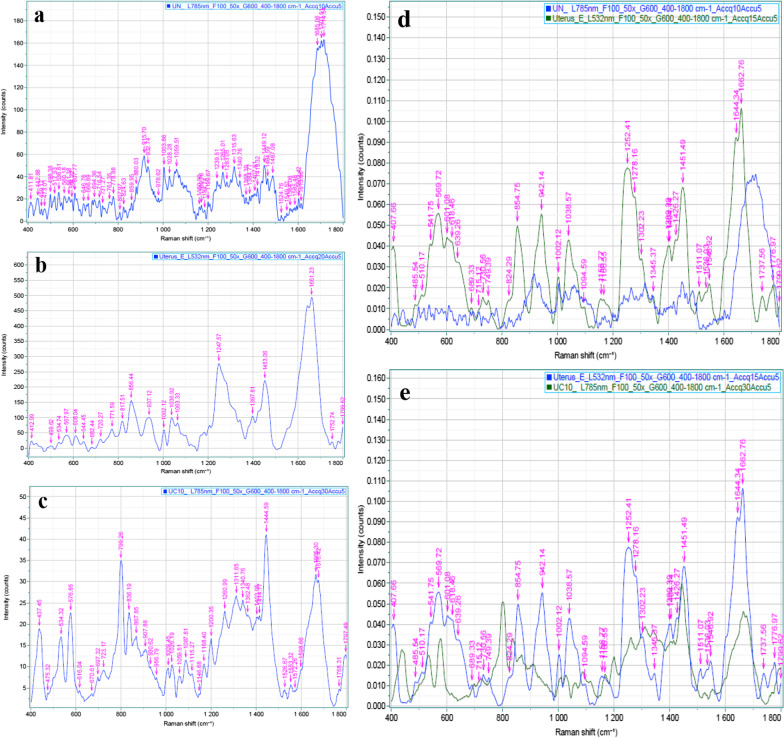
Raman spectra diagrams of **a** normal uterus, **b** decellularized uterus, **c** recellularized uterus after 10 days. Comparison spectra diagrams between normal uterus (blue) and decellularized uterus (green) (**d**), and decellularized (green) and recellularized (blue) uterus after 10 days (**e**)

The Raman spectra in normal uterine scaffolds showed a pattern of intense bands for collagen type I or its components at 1660–1680 cm^−1^. There are some small bands for collagen components such as tyrosine and phenylalanine or other amides at 1302, 1278, 1265, 1239, 1002 and 859 cm^−1^. The spectra of nucleic acid contents including guanine and thymine DNA bases, ribose of RNA mode, and DNA showed intense bands at 666, 1315, 915 and 1094 cm^−1^ respectively. Polysaccharides' skeletal mode showed a band at 942 cm^−1^ [10]. The lipid components showed an intense band at 1449 and 1302 cm^−1^ (Fig. 6a). The Raman spectra in decellularized uterine scaffold showed a sharp peak at 1661 cm^−1^ for collagen components. There is an intense band at 1453 cm^−1^ that corresponding to CH_2_/CH_3_ groups is associated with elastin and collagen. The amide III showed a band at 1247 cm^−1^ assigned to collagen. Three bands at 937, 856 and 817 cm^−1^ assigned to proline and hydroxyproline of collagen type I. There is no band to approve DNA residue in the decellularized uterine scaffold (Fig. 6b). The Raman spectra in the recellularization uterine scaffold showed intense bands for amide III at 1678 and 1665 cm^−1^. There is a sharp band for cholesterol saw at 1444 cm^−1^. The spectra showed some recorded bands between 1120 and 1200 cm^−1^ and at 1311 cm^−1^ that revealed DNA, RNA, phosphate groups, and phosphatidylinositol and guanine. Also, two bands at 576 and 799 cm^−1^ referred to phosphatidylinositol and phosphate groups respectively (Fig. 6c). The comparison between Raman spectra of normal and decellularized uterine scaffolds confirmed the chemical similarity of the two samples. It showed a similar band pattern of collagen type I including intense bands at 1660 cm^−1^ and the other bands that assigned proline and hydroxyproline and phenylalanine or corresponding to amide I and III [10]. The analysis of decellularized uterine scaffolds confirmed the lack of Raman bands typical of cellular material (Fig. 6d). There are Raman bands typical of cellular material in recellularized uterine scaffolds compared to the decellularized uterine scaffolds (Fig. 6e).

## Discussion

Women's menstrual blood as a new source of adult stem cells (MenSCs) is a good choice for cell therapy [5, 11, 12]. Decellularized organs provide an appropriate ECM for tissue-specific cell differentiation. The uterus, as a necessary organ for passing on the stages of embryonic development, is a special option for tissue engineering. Therefore, in this study, we tested the efficiency of cultured MenSCs in the uterine decellularized scaffold. SDS and Triton X-100 were selected for uterine decellularization. Detergents such as SDS have detrimental effects on the ECM constituents. SDS as an anionic detergent with a polar head at the end of the hydrocarbon chain is a hydrophobic (amphipathic) structure. It interacts with proteins and performs decellularization using the destruction of the cell membrane and nucleus. Triton X-100 as a nonionic detergent has a hydrophilic polyethylene oxide chain and hydrophobic group. This detergent eliminates interaction between lipids and lipid-proteins but does not affect protein interaction, which is useful for maintaining extracellular matrix structure [13]. The data from the current study indicated SDS and Triton X-100 could decellularize the uterus completely. The hoechst staining of decellularized scaffold confirmed the absence of cells. This dye binds to adenine–thymine (A–T) regions of the minor groove of DNA and exhibits distinct fluorescence emission spectra that are dependent on dye-base pair ratios. The complete decellularization of rabbit lung [14], monkey kidneys [15] has been reported by SDS and Triton X-100. The introduced decellularization protocol could be employed in future in vitro and in vivo studies.

Adult stem cells are derived from various tissues such as bone marrow, adipose tissue, umbilical cord blood, and amniotic fluid, but due to difficult access, invasive methods of isolation from individuals, and in some cases, the power of proliferation and low differentiation, the use of these cells also have been constrained. Based on therapies in regenerative medicine hundreds of clinical trials of various MSC have overall shown underwhelming results in regenerative medicine applications. Considerable research has been done on the extraction and differentiation of stem cells of menstrual blood into different cell types in tissue engineering research [5].

Partial toxicity of the ficoll in indirect culture method of MenSCs isolation caused the number of stem cells to were lesser than the direct culture method. Blood clots rich in nutrients and fibrin, the absence of substances such as ficoll and antibiotics could increase the number of cells in the direct culture method. In direct culture, the number of stem cells obtained is three times as high as the ficoll separation method [7]. MenSCs were used to treat liver fibrosis in mice [16], improved skin ulcers by differentiation into keratinocytes [17] and treat and improve premature ovarian failure (POF) in the mouse model [18, 19].

Age of donor affects many of mesenchymal stem cells functions such as anti-apoptotic mechanisms and proliferation rate, all of which reduce with aging [20]. However, the proliferative capacity of MenSCs does not appear donor dependent in individuals aged up to 40 years. Older donors (> 40 years) showed a weaker proliferation rate of MenSCs [21]. Highly passaged cells revealed increasing in size, losing fibroblastic morphology, and appearing senescent. Pluripotency protein expression reduced during passaging of MenSCs, from 97.5% at the first passage to 19.4% at passage twelve [22].

MTT assay showed a significant difference in the viability of MenSCs seeded on the decellularized scaffolds after 10 days cultures compared to 7 days cultures and the cells could survive properly within the uterine decellularized scaffolds. The ECM has chemical properties to trigger mitotic activity in the MenSCs which is critical for tissue reconstruction [23].

The results of the electron microscope of the scaffold showed that the three-dimensional structure of the ECM was well preserved. Immunohistochemical staining confirmed the MenSCs penetrated to the uterine scaffold and differentiated to the different cells of the normal uterus such as epithelial cells and smooth muscles. ECM is an essential factor for differentiation, cellular behavior, and gene regulation [24]. The MenSCs nesting and proliferation have been reported in the presence of induction agents in the porous silicon scaffold matrix of cardiac cells [25]. The MenSCs differentiated to hepatocytes on a three-dimensional nanofiber scaffold [26]. MenSCs show considerable capacity to differentiate into numerous lineages in vitro, although have lesser ability for several MSC lineages. It would now be important to demonstrate the potential of MenSCs to undergo differentiation into these lineages in vivo in animal models. It was shown that MenSCs influence the humoral immune responses in a mouse model of heart transplantation by attenuating antibody responses [27].

The recovery properties of recellularized scaffolds are assessed by their structure and composition. Therefore, preserved GAGs, proteins, proline, and hydroxyproline affect the ability to regenerate and elasticity. Confocal Raman spectroscopy revealed general trends of spectra for collagen fiber in uterine decellularized and recellularized scaffolds that are closely similar. There are many bands at different spectra regions that are assigned for collagen or its components, for example, amide III, amino acids such as tyrosine, phenylalanine, proline, hydroxyproline, and CH_2_/CH_3_ groups associated with elastin and collagen. Despite minor differences, Raman spectra obtained from cell-loaded scaffolds accommodate the spectra from the intact uterus. These spectra were attributed to the typical DNA and RNA bands and = C–H in phospholipids, amide III, cholesterol phosphate groups, and phosphatidylinositol and guanine. All of these components are present in the cells, which confirms the presence of cells in the scaffolds.

## Conclusions

The complete decellularization from mice uterine tissue was done by combining chemical agents (ionic and non-ionic detergents). In such a way that the structure of the extracellular matrix was well preserved and suitable natural and non-cellular scaffold was created for tissue engineering. MenSCs extraction was more successful with the direct culture method and more cellular population was obtained. Recellularized scaffold examinations confirmed the success of this method in the development of the uterus. The MenSCs after 10 days have nested in the natural pore of the scaffolds. Tissue engineering by this method provides a future vision for regeneration medicine. The outcomes of this study would bring us one step closer to the ultimate goal of whole human uterus bioengineering.

## Data Availability

All data generated and analyzed during this study are included in this published article.

## Abbreviations

CK: Cytokeratin
DAB: Diaminobenzidine
Des: Desmin
DMEM: Dulbecco’s modified Eagle’s medium
ECM: Extracellular matrix
EDS: Energy dispersive X-ray spectrometer
FBS: Fetal bovine serum
H&E: Hematoxylin and Eosin
IHC: Immunohistochemictry
MB: Menstrual blood
MenSCs: Menstrual blood stem cells
MTT: 3-(4, 5-Dimethylthiazolyl-2)-2, 5-diphenyltetrazolium bromide
OCT: Optimal cutting temperature
OD: Optical density
PBS: Phosphate-buffered saline
SDS: Sodium dodecyl sulfate
SEM: Scanning electron microscopy
SMA: Smooth muscle actin
Vim: Vimentin

## References

[1] Barzegar A (2020). Epidemiologic study of traffic crash mortality among motorcycle users in Iran (2011–2017). Chin J Traumatol.

[2] Yang Q (2008). A cartilage ECM-derived 3-D porous acellular matrix scaffold for in vivo cartilage tissue engineering with PKH26-labeled chondrogenic bone marrow-derived mesenchymal stem cells. Biomaterials.

[3] Cui C-H (2007). Menstrual blood-derived cells confer human dystrophin expression in the murine model of Duchenne muscular dystrophy via cell fusion and myogenic transdifferentiation. Mol Biol Cell.

[4] Liu Y (2019). Therapeutic potential of menstrual blood-derived endometrial stem cells in cardiac diseases. Cell Mol Life Sci.

[5] Borlongan CV (2010). Menstrual blood cells display stem cell–like phenotypic markers and exert neuroprotection following transplantation in experimental stroke. Stem Cells Dev.

[6] Mou X-Z (2013). Menstrual blood-derived mesenchymal stem cells differentiate into functional hepatocyte-like cells. J Zhejiang Univ Sci B.

[7] Kazemnejad S, et al. Chondrogenic differentiation of menstrual blood-derived stem cells on nanofibrous scaffolds. Stem Cell Nanotechnol Methods Protoc 2013; 149–169. 10.1007/7651_2013_9 23592035

[8] Faramarzi H (2016). The potential of menstrual blood-derived stem cells in differentiation to epidermal lineage: a preliminary report. World J Plast Surg.

[9] Bancroft JD, Stevens A. Theory and practice of histological techniques. 1990.

[10] Movasaghi Z, Rehman S, Rehman IU (2007). Raman spectroscopy of biological tissues. Appl Spectrosc Rev.

[11] Allickson J, Xiang C (2012). Human adult stem cells from menstrual blood and endometrial tissue. J Zhejiang Univ.

[12] Patel AN (2008). Multipotent menstrual blood stromal stem cells: isolation, characterization, and differentiation. Cell Transpl.

[13] Crapo PM, Gilbert TW, Badylak SF (2011). An overview of tissue and whole organ decellularization processes. Biomaterials.

[14] Shahri NM (2013). In vitro decellularization of rabbit lung tissue. Cell J (Yakhteh).

[15] Nakayama KH (2010). Decellularized rhesus monkey kidney as a three-dimensional scaffold for renal tissue engineering. Tissue Eng Part A.

[16] Chen L (2017). Human menstrual blood-derived stem cells ameliorate liver fibrosis in mice by targeting hepatic stellate cells via paracrine mediators. Stem Cells Transl Med.

[17] Akhavan-Tavakoli M (2017). In vitro differentiation of menstrual blood stem cells into keratinocytes: a potential approach for management of wound healing. Biologicals.

[18] Liu T (2014). Transplantation of human menstrual blood stem cells to treat premature ovarian failure in mouse model. Stem Cells Dev.

[19] Wang X-J (2017). Human menstrual blood-derived mesenchymal stem cells as a cellular vehicle for malignant glioma gene therapy. Oncotarget.

[20] Alt E (2012). Aging alters tissue resident mesenchymal stem cell properties. Stem Cell Res.

[21] Chen J (2015). Effects of donors’ age and passage number on the biological characteristics of menstrual blood-derived stem cells. Int J Clin Exp Pathol.

[22] Khanmohammadi M (2014). Modified protocol for improvement of differentiation potential of menstrual blood-derived stem cells into adipogenic lineage. Cell Prolif.

[23] Reing JE (2009). Degradation products of extracellular matrix affect cell migration and proliferation. Tissue Eng Part A.

[24] Everitt EA, Malik AB, Hendey B (1996). Fibronectin enhances the migration rate of human neutrophils in vitro. J Leukoc Biol.

[25] Rahimi M (2014). Evaluation of menstrual blood stem cells seeded in biocompatible Bombyx mori silk fibroin scaffold for cardiac tissue engineering. J Biomater Appl.

[26] Sani F (2016). Differentiation of menstrual blood derived stem cell (MensSCs) to hepatocyte-liked cell on three dimensional nanofiberscaffold: poly caprolacton (PCL). J Biomed Sci Eng.

[27] Xu X (2017). Prolongation of cardiac allograft survival by endometrial regenerative cells: focusing on B-cell responses. Stem Cells Transl Med.

